# Personal

**Published:** 1916-07

**Authors:** 


### PERSONAL.

**Announcement of removal, travel, and other matters of interest are requested. Please report errors in the listing of any physician in the State and other directories, that we may co-operate with the State Society in securing a correct list.**

---

Dr. N. Victoria Chappell and Miss E. Mae Chappell of Buffalo left June 8 for a trip to Colorado.

---

Dr. David E. Wheeler, formerly of Buffalo, has recently been honorably retired from the French Army. It will be remembered that he served as surgeon in the Foreign Legion and was wounded in the leg, while bringing in the wounded under fire. He was specially mentioned for heroism on the field of battle, in the day's despatches.

---

Dr. Alfred S. Burdick of Chicago, of the editorial staff of The American Journal of Clinical Medicine, has been appointed Assistant General Manager of the Abbott Laboratories.

---

Dr. J. C. Partridge of North Cohocton, has moved to Avoca.

---

Dr. Frank Trippe, University of Buffalo, 1916, was tendered a reception at the Club of St. Anthony's Beneficial Society, in Fredonia, shortly after his graduation.

---

John Opie McCall, D. D. S., announces the removal of his offices to 437 Franklin Street, Buffalo. Practice limited to Oral Prophylaxis, Periodontia and Consultation on Mouth Infections.

---

Dr. John R. Butsch of Buffalo, has been appointed to the staff of the Mayo Clinic at Rochester, Minn. In his absence, his laboratory will be continued at 519 Franklin Street, under the direction of Dr. R. R. Donk.

---

Dr. Wm. A. Curtin of Syracuse has purchased the residence of the late Henry L. Elsner, adjoining his own property of Fayette Park.

---

Robert Murray, D. D. S., has been elected President of the Dental Society of N. Y. State.

---

Dr. F. M. Evans has been appointed Post Master of Fredonia.

---

Dr. John H. Evans of Buffalo has moved to 519 Franklin Street.

---

Dr. Francis J. Lennon of Buffalo leaves July 1 to spend some weeks at his parents' summer home at Rockaway Beach, L. I.

---

Dr. Douglas P. Arnold of Buffalo has moved to 758 Elmwood Avenue.

---

Dr. Franklin W. Barrows has been appointed Acting Chief of the Bureau of Child Hygiene of Buffalo, vice Dr. Arthur C. Schaefer, who has been called into service with the 74th Regiment of the National Guard.

---

Dr. Herman D. Andrews of Buffalo announces the removal of his office to Suite 15, 494 Franklin Street, Hours 9-1.

